# How countries can reduce child stunting at scale: lessons from exemplar countries

**DOI:** 10.1093/ajcn/nqaa153

**Published:** 2020-07-21

**Authors:** Zulfiqar A Bhutta, Nadia Akseer, Emily C Keats, Tyler Vaivada, Shawn Baker, Susan E Horton, Joanne Katz, Purnima Menon, Ellen Piwoz, Meera Shekar, Cesar Victora, Robert Black

**Affiliations:** Centre for Global Child Health, Hospital for Sick Children, Toronto, Ontario, Canada; Dalla Lana School of Public Health, University of Toronto, Toronto, Ontario, Canada; Center of Excellence in Women and Child Health, the Aga Khan University, Karachi, Pakistan; Centre for Global Child Health, Hospital for Sick Children, Toronto, Ontario, Canada; Centre for Global Child Health, Hospital for Sick Children, Toronto, Ontario, Canada; Centre for Global Child Health, Hospital for Sick Children, Toronto, Ontario, Canada; United States Agency for International Development, Washington, DC, USA; School of Public Health and Health Systems, University of Waterloo, Waterloo, Ontario, Canada; Johns Hopkins Bloomberg School of Public Health, Baltimore, MD, USA; Poverty, Health and Nutrition Division, International Food Policy Research Institute, South Asia Office, New Delhi, India; Global Development Division, Bill & Melinda Gates Foundation, Seattle, WA, USA; Health, Nutrition & Population, World Bank, Washington, DC, USA; Federal University of Pelotas, Pelotas, Rio Grande do Sul, Brazil; Johns Hopkins Bloomberg School of Public Health, Baltimore, MD, USA

**Keywords:** stunting, linear growth, children, nutrition, exemplar, mixed methods

## Abstract

**Background:**

Child stunting and linear growth faltering have declined over the past few decades and several countries have made exemplary progress.

**Objectives:**

To synthesize findings from mixed methods studies of exemplar countries to provide guidance on how to accelerate reduction in child stunting.

**Methods:**

We did a qualitative and quantitative synthesis of findings from existing literature and 5 exemplar country studies (Nepal, Ethiopia, Peru, Kyrgyz Republic, Senegal). Methodology included 4 broad research activities: *1*) a series of descriptive analyses of cross-sectional data from demographic and health surveys and multiple indicator cluster surveys; *2*) multivariable analysis of quantitative drivers of change in linear growth; *3*) interviews and focus groups with national experts and community stakeholders and mothers; and *4*) a review of policy and program evolution related to nutrition.

**Results:**

Several countries have dramatically reduced child stunting prevalence, with or without closing geographical, economic, and other population inequalities. Countries made progress through interventions from within and outside the health sector, and despite significant heterogeneity and differences in context, contributions were comparable from health and nutrition sectors (40% of change) and other sectors (50%), previously called nutrition-specific and -sensitive strategies. Improvements in maternal education, maternal nutrition, maternal and newborn care, and reductions in fertility/reduced interpregnancy intervals were strong contributors to change. A roadmap to reducing child stunting at scale includes several steps related to diagnostics, stakeholder consultations, and implementing direct and indirect nutrition interventions related to the health sector and nonhealth sector .

**Conclusions:**

Our results show that child stunting reduction is possible even in diverse and challenging contexts. We propose that our framework of organizing nutrition interventions as direct/indirect and inside/outside the health sector should be considered when mapping causal pathways of child stunting and planning interventions and strategies to accelerate stunting reduction to achieve the 2030 Sustainable Development Goals.

## Introduction

The global prevalence of childhood linear growth retardation, commonly measured as stunting, has declined over the last decades, but remains unacceptably high. In most cases, such growth failure is seen in the wake of persistent and widespread poverty, chronic undernutrition, and adverse environmental exposures. The bulk of linear growth faltering seen in children aged <5 y occurs within 1000 d after conception (1, 2). It can start in the womb, in the context of poor maternal nutrition and infection, and extends to the first 2 y of life and sometimes beyond (3). The 2012 World Health Assembly (WHA) endorsed a 40% reduction in the number of stunted under-5 children by 2025 (4), a commitment that was reinforced by target 2.2 of the Sustainable Development Goals (SDGs), which explicitly outlines the global pledge to reduce the prevalence of child stunting. Despite these longstanding targets, we are not on track to reach them (5).

Previous *Lancet* series have highlighted the importance of optimizing childhood nutrition for survival (6, 7), early child development, and human capital development across the life course (8). These series also underscore many evidence-based and cost-effective interventions that can improve maternal and child nutritional status and reduce morbidity and mortality (9–11). Since, new evidence has been generated, including the impacts of lipid-nutrient supplements on stunting, wasting, anemia (12). Much of the evidence in relation to the potential impact of interventions comes from modest-scale randomized trials and meta-analyses of effect sizes in varying contexts. The 2013 *Lancet* maternal and child nutrition series estimated that scaling up a set of 10 evidence-based “nutrition-specific” interventions to 90% coverage in 34 focus countries with 90% of the global stunting burden could result in a 20.3% (10.2–28.9%) stunting reduction (10). Econometric analyses done by Smith and Haddad in 2015 (13) and Headey in 2013 (14) also sought to quantify the effect of changes in basic and underlying determinants on population-level stunting reductions, and highlighted the importance of agricultural productivity, gender equality, women's education, water and sanitation infrastructure, health services access, and fertility rates—the targets of “nutrition-sensitive” interventions.

The dearth of population-based data on dietary intake has made assessing the contribution of nutrient adequacy, an immediate determinant of linear growth retardation, difficult. In addition, there are limited data on programmatic coverage of high-impact, nutrition-specific interventions. Multiple contributing factors mean that policies, programs, and interventions to drive stunting reduction must involve different sectors and delivery platforms (15). The determinants and risks associated with concurrent stunting and wasting also merit examination, because linear growth faltering is not the only issue affecting child well-being in vulnerable populations (16–18).

There are a range of overlapping, hierarchical, and potentially intergenerational determinants along the causal chain to poor child growth and stunting that can contribute differently to outcomes in various contexts. The first article of this supplement reviewed the major drivers that have led to national reductions in stunting prevalence, operating at basic, underlying, and immediate levels in >70 countries, the majority of which are low- and middle-income countries (LMICs) (19). Determinants of particular importance were improved parental literacy rates, household socioeconomic status, water, sanitation, and hygiene (WASH) conditions, health services access, and family planning. Recent case studies of success have identified these same factors as contributing to change in undernutrition over time in a set of LMICs (20). These studies also highlighted the role of several factors that enabled policy commitments to nutrition and program strategies to scale up nutrition interventions (20).

In an effort to expand, strengthen, and contribute to the current knowledge base, this article consolidates findings from our series of systematic mixed-methods exemplar case studies with the aim of providing summative guidance on how to accelerate reductions in national stunting prevalence in order to achieve our WHA and SDG targets. Exemplar countries are those that have an outsized reduction in stunting prevalence in a 15–20-y period relative to economic growth (as defined earlier in the “Methods” article of this supplement) (21) and include Peru, Kyrgyz Republic, Nepal, Ethiopia, and Senegal as examples from Latin America, Central Asia, South Asia, East Africa and West Africa, respectively. Specifically, we *1*) review the current burden, trends, and distribution of child stunting prevalence in exemplar countries; *2*) contrast key basic, underlying, and immediate drivers of child stunting reduction across exemplar countries; *3*) undertake an assessment of policy and programmatic investments across exemplars; *4*) propose a framework for categorizing drivers of change; and *5*) suggest a roadmap for national or subnational efforts to reduce child stunting at scale.

## Methods

Data and evidence for this article were drawn from our country case studies that were undertaken using mixed methods as described in Paper 2 of this series (21). As detailed in the article, our approach was informed by existing literature and published methodological approaches to study success stories (22). Initially, a systematic literature review of more than 15 published peer-reviewed and gray literature sources across all LMICs with an expanded search for exemplar countries was undertaken. Studies published between 1990 and 2018 were screened for relevance and synthesized. The aim was to identify and synthesize information on contextual factors, national and subnational interventions, policies, strategies, programs, and initiatives that might have theoretically contributed to stunting reduction in each country over time. Findings from this exercise were used to inform the research process and methodology, and support findings and inferences.

The final set of methods included: *1*) a series of descriptive analyses of cross-sectional data; *2*) a deterministic analysis of quantitative drivers of change in linear growth; *3*) country-level stakeholder interviews; *4*) a review of policy and program evolution over the time period of study; and *5*) an integrative analysis of insights from these 4 core research activities.

**FIGURE 1:**
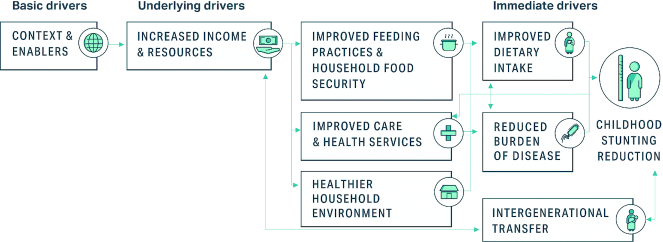
Conceptual framework for analyzing determinants of child nutrition. Adapted from references 21, 6, and 7.

1. A series of descriptive analyses were undertaken to explore levels, distributions, and trends in the main outcomes examined, that is, child height-for-age *z*-scores (HAZ) and under-5 child stunting prevalence (HAZ < −2 SD of the WHO growth reference standards). Using cross-sectional data, we examined stunting prevalence geospatial patterns and inequalities by key dimensions (wealth quintiles, maternal education, urban/rural residence, and child gender). Kernel density plots of child HAZ scores were examined to understand the population HAZ shifts over time. Child age compared with HAZ growth trajectories (Victora curves) were fitted using smoothed polynomial regressions to study stunting risk at birth and how the growth faltering process changes with age.
2. Using household survey datasets (e.g., demographic and health surveys, multiple indicator cluster surveys) and various country-level administrative data sources, we assembled cross-sectional panels into a pseudo-cohort design to study multivariable quantitative drivers of child stunting reduction in each country using Oaxaca–Blinder decomposition methods and linear mixed-effects regression with a difference-in-difference design.
3. We conducted in-depth interviews with national experts from within and outside the health sector (10–20 participants in each country), in-depth interviews with community-level stakeholders (10–15 participants), and focus group discussions with mothers in communities (2–4 focus groups in each country with 10–15 participants each) to understand the macro (policy, programs, contextual factors) and micro (individual, household, community) factors that contributed to stunting reduction.
4. A comprehensive review of programs/policies/strategies/laws/legislation adopted from the 1990s (or earlier) to 2018 from within and outside the health sector was undertaken to identify key national investments and efforts that each country adopted to reduce stunting at scale. Detailed methods for this review are included in Paper 2 of this series (21).
5. Results from each research activity were analyzed iteratively and shared with technical experts, research partners, and research leads to develop a strong evidence-based narrative of stunting reduction drivers for exemplar countries. The conceptual framework for improved nutrition proposed by UNICEF (23) and adapted by Black et al. (6, 7) (2008, 2013) was used to guide all analyses and inferences (**Figure 1**). Analysis in this article consolidates and synthesizes literature, policy/program, qualitative, and quantitative findings across exemplar countries.

## Results

### Descriptive trends

**FIGURE 2:**
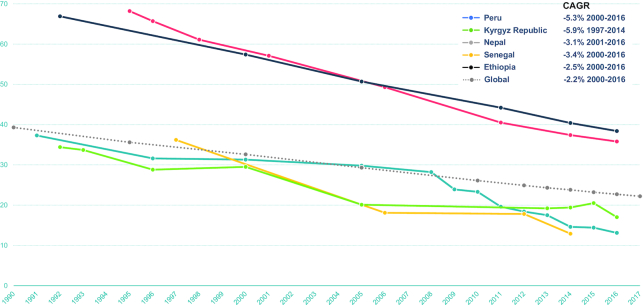
Trends in child stunting prevalence in exemplar countries (1990–2016). CAGR, compound annual growth rate.

Stunting prevalence baselines in exemplar countries generally cluster into 2 groups (**Figure 2**), those that start at 50–60% baselines (Nepal, Ethiopia) and those with 30–40% baselines (Peru, Kyrgyz Republic, Senegal), and compound annual growth rates range from −2.5% to −5.9% in the ∼2000–2016 period. Peru (−5.3%) and Kyrgyz Republic (−5.9%) have almost double the rate of reduction of other countries. Similar progress in stunting reduction across exemplar nations is impressive considering the diverse contextual starting points for each country (**Table 1**). Population size and economic growth vary considerably; for instance, Kyrgyz Republic had only 6.1 million citizens in 2016 whereas Ethiopia had a total population of 102.4 million. All countries saw continued economic growth in the study period (range 3.3–8.2%/y) although their gross domestic product per capita varied vastly in 2016 ($1746 for Ethiopia compared with $13,404 for Peru, purchasing power parity (PPP) measured in current international dollars). Despite differences, across exemplar countries, gains in overall development, employment, literacy, female empowerment, household conditions, health and out-of-pocket spending, health worker availability, and maternal and child health (among other indicators) have been observed (Table 1).

**TABLE 1:** Trends in demographic, economic, and other contextual factors for exemplar countries^1^

	Nepal			Peru			Senegal			Kyrgyz Republic			Ethiopia		
Indicator	2000	2016	CAGR, %	2000	2016	CAGR, %	1992	2017	CAGR, %	2000	2016	CAGR, %	2000	2016	CAGR, %
Total population (millions)	23.7	29.0	1.3	25.9	31.8	1.3	8.0	15.9	2.8	4.9	6.1	1.4	66.5	102.4	2.7
Population growth (annual %)	1.9	1.1	−3.0	1.4	1.3	−0.5	3.0	2.8	−0.3	1.2	2.1	3.6	2.9	2.5	−0.9
Urban population (% of total)	13.4	19.0	2.2	73.0	77.5	0.4	39.2	46.7	0.7	35.3	35.9	0.1	14.7	19.9	1.9
Human Development Index	0.446	0.558 (2015)	1.5	0.677	0.74 (2015)	0.6	0.37 (1995)	0.51	1.5	0.593	0.664 (2015)	0.8	0.283	0.457	3.0
Households with piped water access, %	35.4 (2001)	51.4	2.5	72.3	78.9 (2012)	0.7	46.8	68.8	1.6	74.5 (1997)	82.1 (2012)	0.7	17.6	32.0	3.8
Open defecation (% engaging in)	66.9	24.0	−6.2	18.3	7.1	−5.8	38.9	14.9	−3.8	0.1	0	−100.0	79.1	25.6	−6.8
GDP per capita, PPP (current international $)	1215.0	2642.0	5.0	5115.1	13,404.2	6.2	1559.2	3556.1	3.4	1650.8	3567.8	4.9	494.3	1746.2	8.2
GNI per capita, PPP (current international $)	1220.0	2680.0	5.0	5000.0	12,850.0	6.1	1530.0	3460.0	3.3	1550.0	3380.0	5.0	490.0	1740.0	8.2
Poverty headcount ratio at $1.90/d (2011 PPP) (% of population)	14.5 (2003)	3.1 (2010)	−19.8	16.7	3.0 (2015)	−10.8	68.4 (1991)	38.0 (2011)	−2.9	42.2	2.5 (2015)	−17.2	61.2 (1999)	27.3 (2015)	6.2
Total unemployment (% of total labour force)	1.8	3.1	3.5	7.3	6.7	−0.5	5.7 (2002)	6.9 (2015)	1.6	7.5	7.2	−0.3	3.7 (1999)	2.3 (2013)	6.7
Total health expenditure (% of GDP)	3.6	6.2 (2015)	3.7	4.8	5.5 (2014)	0.9	4.6 (2000)	3.9 (2015)	−1.0	4.7	6.5 (2014)	2.3	4.4	4.0	−0.6
Public health expenditure (% of GDP)	1.4	2.3 (2014)	3.6	2.7	3.3 (2014)	1.4	36.7 (2000)^2^	31.7 (2015)	−1.0	2.1	3.6 (2014)	3.9	41.2^2^	27.6	−2.5
Out-of-pocket health expenditure (% of total health expenditure)	55.8	60.4 (2014)	0.6	36.4	28.6 (2014)	−1.7	54.0 (2000)	44.2 (2015)	−1.3	49.8	39.4 (2014)	−1.7	36.0	37.4	0.2
Net ODA received (current US$) (millions)	386.4	1065.9	6.6	401.6	331.8	−1.2	662.4	736.4 (2016)	0.7	214.7	769.0	8.3	10.3	39.8	8.8
Age at first marriage (median, women aged 20–49 y)	17.0 (2001)	18.1	0.4	21.4	21.6 (2012)	0.08	16.6	19.7 (2015)	1.2	20.4 (1997)	20.6 (2012)	0.07	16.4	17.5	0.4
Child marriage (% women 20–24 y married by age 18)	56.1 (2001)	39.5	−2.3	18.7	18.6 (2014)	−0.04	47.7	31.5 (2016)	−2.6	21.2 (1997)	11.6 (2014)	−3.5	49.1	41.0 (2011)	−1.2
Fertility rate (average births per woman)	4.0	2.1	−3.9	2.9	2.4 (2015)	−1.3	6.0	4.6	−1.1	2.4	3.2 (2015)	1.9	5.9	4.6	−1.5
Births attended by skilled health staff (% of total)	11.9	58.0	10.4	59.0	90.0 (2014)	3.1	47.2	68.4	1.5	98.6	98.4 (2014)	−0.01	5.6	15.5 (2014)	7.5
Antenatal care ≥4 visits (% of mothers)	14.3 (2001)	69.4 (2016)	11.1	68.5	94.4 (2012)	2.7	14.0	57.0	5.8	No data	83.6 (2012)	N/A	10.0	32.0	7.5
Adolescent fertility rate (births/1000 girls aged 15–19 y)	113.9	62.1	−3.7	65.0	48.0 (2015)	−2.0	127.0	78.0	−1.9	45.9	39.2 (2015)	−1.1	110.1	64.9	−3.3
Adult literacy rate (% of adults aged ≥15 y)	48.6 (2001)	59.6 (2011)	2.1	87.7 (2004)	94.2	0.6	39.3 (2002)	51.9	1.9	no data	99.2 (2009)	N/A	35.9 (2004)	39.0 (2007)	2.8
Female adult literacy rate (% of females aged ≥15 y)	34.9 (2001)	48.8 (2011)	3.4	82.1 (2004)	91.2	0.9	29.3 (2002)	39.8	2.1	no data	99.0 (2009)	N/A	22.8 (2004)	28.9 (2007)	8.2
Female youth literacy rate (% of females aged 15–24 y)	60.1 (2001)	80.2 (2011)	2.9	95.7 (2004)	98.7	0.3	41.0 (2002)	63.5	3.0	no data	99.8 (2009)	N/A	38.5 (2004)	47.0 (2007)	6.9
Gender Inequality Index (0–1; closer to 1 is higher inequality)	0.670	0.497 (2015)	−2.0	0.440 (2005)	0.385 (2015)	−1.3	0.64 (1995)	0.52	−0.9	0.476	0.394 (2015)	−1.3	0.6 (2005)	0.5	−1.6
Gender Development Index	0.769	0.925 (2015)	1.2	0.921	0.949	0.2	0.781 (1995)	0.911	0.7	0.959	0.960	0.01	0.7	0.9	1.6
Density of physicians (per 1000)	0.052 (2001)	0.598 (2014)	20.7	1.166 (1999)	1.116 (2012)	−0.3	0.07	0.07 (2016)	0.0	2.8	1.9 (2014)	−2.7	0.021	0.022 (2010)	0.5
Density of nurses and midwives (per 1000)	0.469 (2004)	2.041 (2014)	15.8	0.669 (1999)	1.299 (2012)	5.2	0.3 (2004)	0.31 (2016)	0.3	5.5 (2008)	6.0 (2013)	1.8	0.215 (2003)	0.236 (2010)	1.3
Child wasting, %	11.3 (2001)	9.7	−1.0	1.1	1.0	−0.6	9.0	7.2 (2016)	−0.9	2.9	2.8	−0.2	12.4	9.9	−1.4

1 Sources: World Bank, United Nations Development Programme, Oxford Poverty and Human Development Initiative, Food and Agriculture Organization of the United Nations. CAGR, compound annual growth rate; GDP, gross domestic product; GNI, gross national income; N/A, not applicable; ODA, official development assistance; PPP, purchasing power parity.

2 This indicator is domestic general government health expenditure (percentage of current health expenditure).

**FIGURE 3:**
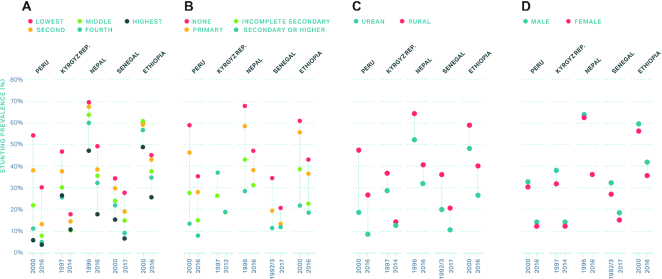
(A) Child stunting prevalence by wealth in exemplar countries. (B) Child stunting prevalence by maternal education in exemplar countries. (C) Child stunting prevalence by residence in exemplar countries. (D) Child stunting prevalence by child sex in exemplar countries.

Strong stunting reduction was coupled with entire child HAZ distribution shifts (see **Supplemental Figure 1** for HAZ kernel density plots) in exemplar countries, suggesting that child growth faltering is reducing at a national level. Examining kurtosis is useful to measure outliers in the data, with higher values indicating fewer outliers (or fewer children in the tails of the HAZ distribution). Kurtosis increased notably over time in Peru and Kyrgyz Republic, suggesting a potential reduction in HAZ inequalities (fewer children in the tails of the distribution) whereas in Senegal, Ethiopia, and Nepal it stayed about the same or even increased. Of course, reduced kurtosis could also indicate better measurement or precision around anthropometry metrics over time, and the difference is difficult to ascertain from HAZ kernel density curves alone (**Supplemental Table 1**). Our equity analysis of stunting prevalence provides further insights into inequalities. Stunting prevalence reduced for all wealth quintiles in Peru (with only the poorest lagging behind) from 2000 to 2016, whereas in Kyrgyz Republic the >20% point gap between the richest and poorest in 1997 became negligible in 2014 (**Figure 3**A). Interestingly, wealth inequalities did not reduce, and in fact, even widened in Nepal, Senegal, and Ethiopia over time. Child stunting prevalence was consistently higher in the least educated mothers, though gaps reduced over time in most exemplar countries (Figure 3B). Families living in rural areas had higher stunting prevalence in all countries, though disparities between urban and rural households did reduce in most countries except Ethiopia, where gaps widened from 2000 to 2016 (Figure 3C). Our assessment across child gender (Figure 3D) revealed minimal differences between boys and girls in general; however, boys were always slightly more stunted than girls in all countries.

**FIGURE 4:**
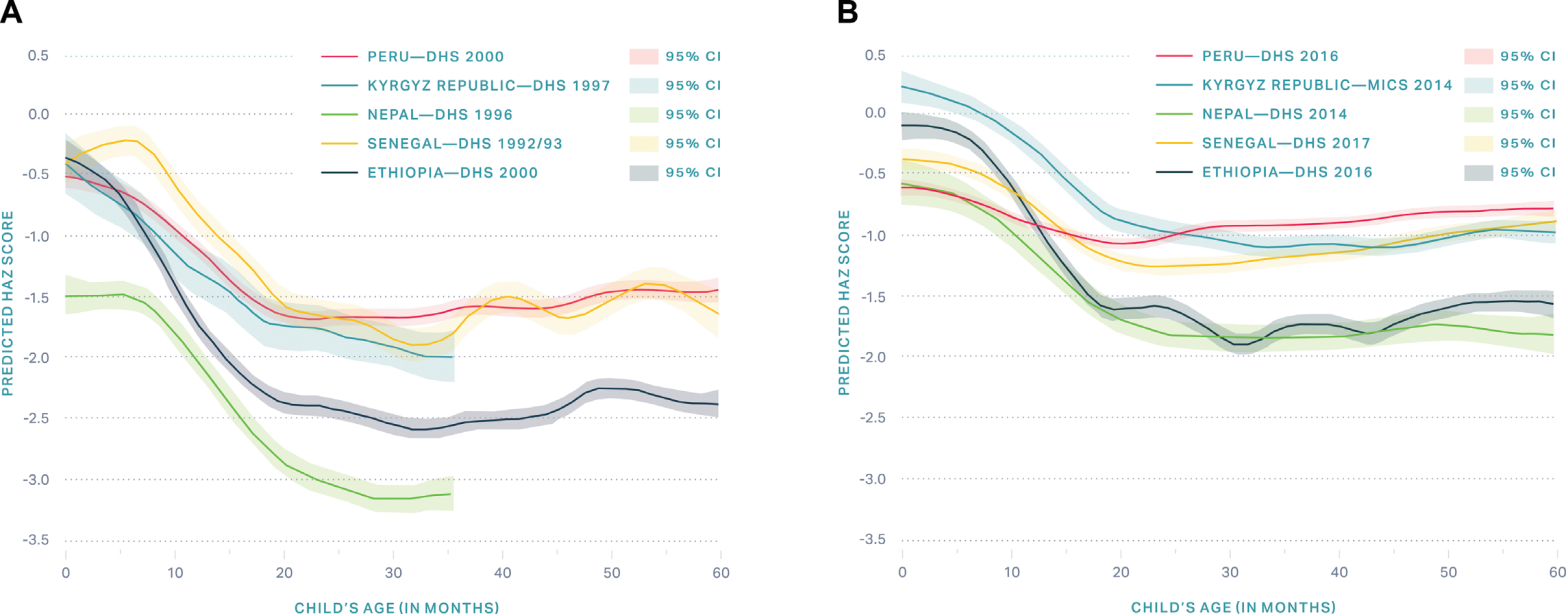
Child height-for-age *z*-score (HAZ) compared with age curves in exemplar countries for earlier period of study (A) and later period (B). DHS, demographic and health survey; MICS, multiple indicator cluster survey. Baseline surveys for Nepal and Kyrgyz Republic collected anthropometry for children younger than 3 years only.

Child HAZ compared with age curve analysis (**Figure 4**) suggests that Ethiopia, Nepal, and Kyrgyz Republic have had substantial improvements in length at birth (i.e., mean child HAZ at *y*-intercept increased and became closer to the global reference median of 0). This indicates that newborns became substantially larger at birth over this time, and could be indicative of improvements in maternal nutritional status (for instance, during adolescence and/or prior to conception) and maternal and newborn care. Peru and Senegal did not make progress in reducing the birth disadvantage. Flattening of the Victora curve between 0 and 6 mo was observed in all countries over time, and could be related to better breastfeeding practices and overall environment for the baby. For children aged 6–23 mo, Peru and Senegal had dramatic flattening of the HAZ curve over time (or slowing of growth faltering), which suggests that major stunting drivers in these 2 countries could be related to improvements in disease prevention and management, better dietary practices, and improved household environment. Ethiopia, Nepal, and Kyrgyz Republic made comparatively fewer gains in this age group.

### Determinants of child HAZ improvements

**FIGURE 5:**
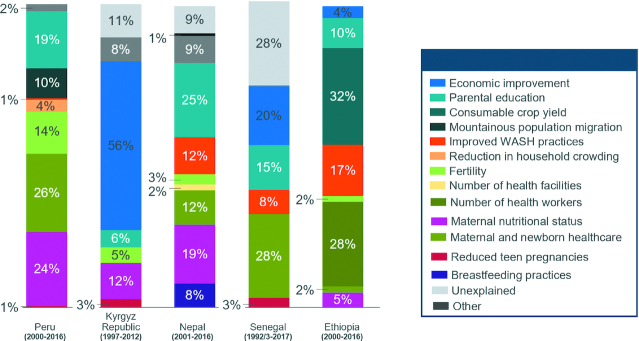
Oaxaca–Blinder HAZ decomposition results for exemplar countries. Note: the Kyrgyz Republic sample is of children aged <3 y. Parental education breakdown: Peru (17.8% maternal, 2.7% paternal), Kyrgyz Republic (5.8% paternal), Nepal (12.2% maternal, 12.5% paternal), Senegal (7.5% maternal, 7.4% paternal), and Ethiopia (5.2% maternal, 5.0% paternal). “Other” category includes child age, gender, and region, The following surveys were excluded due to unreliable data: 2014 Kyrgyz Republic MICS and 1996 Nepal DHS. DHS, demographic and health survey; HAZ, height-for-age *z*-score; MICS, multiple indicator cluster survey ; WASH, water, sanitation, and hygiene.

When examining quantitative drivers of mean HAZ improvement using multivariable Oaxaca–Blinder decomposition (**Figure 5**), our models could explain 72–100% of mean HAZ improvement across countries. Notwithstanding the heterogeneity related to baseline status of health and nutrition indicators and socioeconomic status, the relative contributions of initiatives and factors from within and outside the health sector were evident, and often interventions unrelated to health and nutrition contributed to ∼50% of child stunting reduction (of total child HAZ change over time) in each country. The nonhealth sector–related improvements contributed to 36–70% (median 47%) of the observed changes in child HAZ, whereas health- or health care–related changes contributed to 20–64% of change (median 37%). Some of the gaps related to “unexplained” fractions in Kyrgyz Republic, Senegal, and Nepal are likely related to food security/dietary intake or maternal newborn health care or nutrition improvement because proxies for these were lacking in those countries. Notably, improvements in maternal education, maternal and newborn health care, reduction in fertility/reduced interpregnancy intervals, and maternal nutritional status were strong and common contributors to mean HAZ gains across most countries. Some variations in drivers were evident across the countries examined, a finding that largely resulted from differing country contexts and status at baseline. For example, reduced open defecation (from 79% in 2000 to 26% in 2016) contributed to 17% of the change in HAZ in Ethiopia, whereas improved WASH practices did not emerge as a factor in Kyrgyz Republic because of the highly functional water and sanitation systems and practices that were put in place during the Soviet era. In addition, some indicators included in the decomposition analyses were relevant only to 1 country. In Ethiopia, increased consumable crop yield contributed to 32% of the HAZ improvement whereas in Peru, mountainous population migration accounted for 10%. Although quite different in concept, both indicators could be proxy variables for household food security.

Although we did attempt to include determinants at each hierarchical level of the conceptual framework, these analyses are limited by data availability. Regardless, many of the determinants and strategies that contributed to overall HAZ improvement were consistent across countries.

### Country investments related to stunting reduction

**FIGURE 6:**
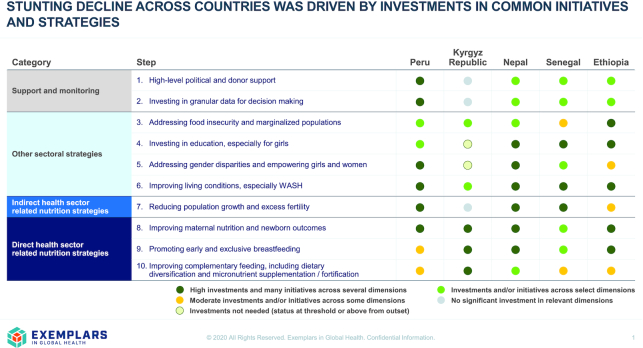
Policy, strategy, and programmatic investments related to stunting reduction in exemplar countries. WASH, water, sanitation, and hygiene.

When examining key policy, strategy, and programmatic investments adopted by countries to reduce child stunting, several commonalities were observed (**Figure 6**). Effective initiatives included health and nutrition interventions addressing immediate determinants that could be grouped into those to improve maternal nutrition and newborn outcomes, promote early and exclusive breastfeeding, and improve complementary feeding practices. In addition, investments in improving reproductive health practices were important for increasing contraceptive use, delaying first pregnancy, and increasing birth spacing. Other sectoral strategies, such as those to improve economic conditions, parental education, and WASH, also played a key role in addressing underlying determinants of child growth. Pivotal to gains were high-level political and donor support as well as sustained financing to improve child health and nutrition overall, and investments in granular data, national capacities for monitoring and decision-making, and capacities for program implementation at scale. These were most prominent in Peru (especially in the Ministry of Finance) but there were also key enablers of stunting reduction in Nepal, Senegal, and Ethiopia (Figure 6).

### A potential new framework for classifying nutrition-related interventions

The 2013 *Lancet* series on maternal and child nutrition was guided by a conceptual framework that outlined the actions needed to achieve optimal child growth and development (7). This framework is divided into nutrition-specific and nutrition-sensitive interventions, programs, and approaches that work to alleviate the immediate and underlying causes of malnutrition, respectively. The framework also outlines the importance of building an enabling environment that will support these strategies through stakeholder buy-in, the generation of rigorous and supportive evidence, advocacy, accountability, coordination, and resource mobilization. Using this approach, nutrition-sensitive programs, such as those relating to agriculture and food security are being leveraged to serve as delivery platforms for nutrition-specific interventions. However, as the evidence base on the impact of nutrition-specific and -sensitive strategies has expanded, and our understanding of what it takes to govern and scale these multisectoral approaches has advanced, the need for further refinement of this framework has become clear.

**FIGURE 7:**
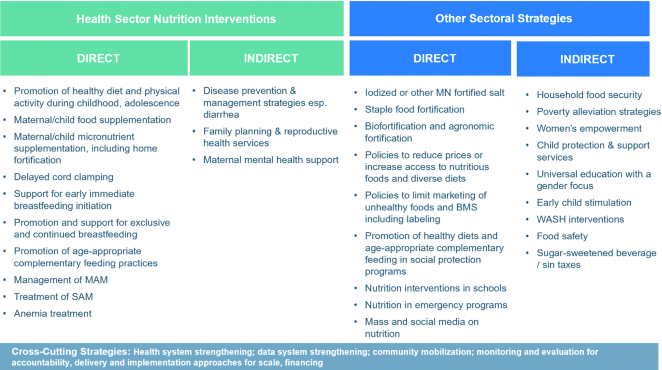
Conceptual framework for interventions related to child and maternal undernutrition. BMS, breastmilk substitutes; MAM, moderate acute malnutrition; MN, micronutrient; SAM, severe acute malnutrition; WASH, water, sanitation, and hygiene.

Building on past efforts (24, 25), we are now proposing that direct or indirect nutrition interventions within the health sector be considered separately alongside other sectoral approaches, the latter working to improve nutrition in a more supportive manner (see **Figure 7**). The direct nutrition interventions, which in this framework extend beyond stunting reduction to encompass multiple nutrition outcomes and conditions such as reduction of wasting and anemia, are nearly synonymous with those that were previously named nutrition-specific. However, several approaches that were previously deemed nutrition-sensitive are better reclassified as indirect nutrition strategies (e.g., disease prevention and management, reproductive health). Other interventions, which also contain direct and indirect actions, fall into the other sectoral strategy category [e.g., agriculture and food security, social safety nets (e.g., conditional cash transfers) and other poverty alleviation strategies, promotion of women's empowerment, child protection, education, WASH]. Within this schema, direct interventions address the immediate determinants of all forms of child undernutrition whereas indirect interventions (that are both inside and outside the health sector) work to alleviate the more distal, underlying determinants. Not all indirect interventions explicitly include a nutrition focus, but sectoral agendas (e.g., related to poverty reduction and improvements in education and sanitation) can firmly and positively address underlying determinants of child growth firmly and positively when implemented with a focus on closing social and geographic equity gaps.

### A roadmap for reducing child stunting at scale

**FIGURE 8:**
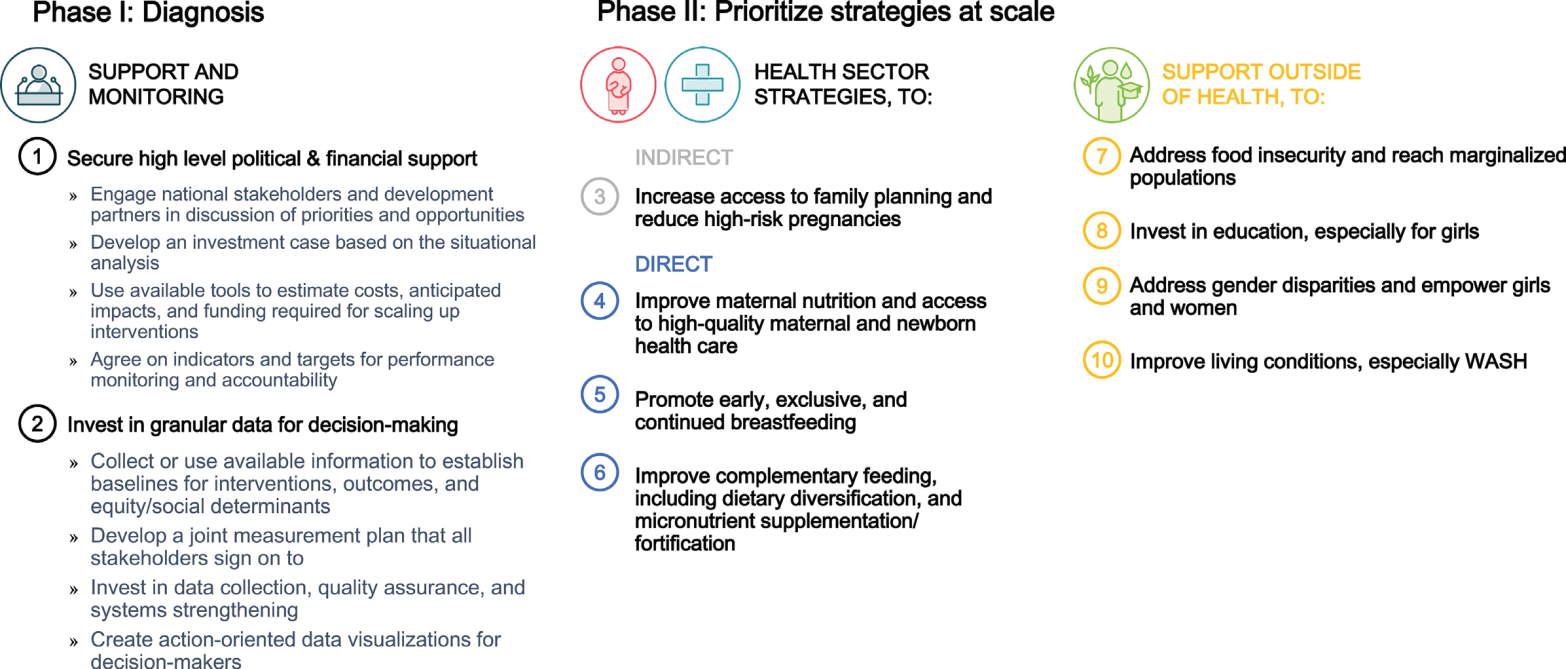
Roadmap to child stunting reduction at scale. NGO, nongovernmental organization; WASH, water, sanitation, and hygiene.

So, what can a country or region do to reduce child stunting at scale? Based on our case study findings and existing evidence, **Figure 8** depicts our proposed roadmap to achieve this end, split into 2 phases. In Phase I, we suggest policy and investment cases for reducing child stunting to begin with a robust diagnostic comprised of a situational analysis and stakeholder consultations. The situational analysis should use both quantitative and qualitative data to create a country-level baseline report of levels, trends, and determinants of child stunting. It would also include an assessment of existing programs and approaches (including new service delivery mechanisms such as results-based approaches, digitally enhanced approaches, and new financing mechanisms such as results-based financing, performance for results, etc.), and a range of accompanying policies, legal acts, and actions in other supporting sectors. A focus on identifying interventions and delivery mechanisms that can ensure equitable reach while addressing capacity and constraints, would be key. For instance, a gap-analysis of what is needed to achieve programmatic scale, including an assessment of financing needs using decision science tools such as Optima Nutrition (26), would be useful.

Findings from the report should be deliberated amongst key stakeholders (e.g., donors, government, nongovernmental organizations, private sector players, etc.) to identify gaps and priorities for different intervention packages and delivery strategies in the health/nutrition sector.

Phase II involves strengthening of delivery systems and implementation of scaled-up actions identified through the iterative process in Phase I in both the health and nutrition sectors and nonhealth sectors. Although we have rank ordered interventions in our framework, each country's adoption of these should depend on its baseline situation, existing status of relevant policies and programs, logistics, capacities, and finances. In addition, policies that shape the broader household and community environment for improvements in child growth require attention such as the extent and nature of economic and agricultural growth, education, and sanitation, with a front and center focus on achieving social equity in these areas. Finally community engagement and buy-in seems critical in many ways. Together, actions across these areas have the potential to accelerate improvements in child growth.

## Discussion

### Summary

This summary article, focused on drivers and initiatives for reducing child stunting at scale, has analyzed global evidence of stunting determinants from LMICs, in-depth case studies from 5 exemplar countries, and the existing nutrition literature base. We found that improvements in child growth faltering and reductions in stunting were related to investments from both within and outside the health sector; we note that ∼40% of the impact came from direct and indirect health-related nutrition strategies, 50% from other sectoral strategies, and 10% was unexplained. Notably, improvements in maternal nutrition, maternal education, broad maternal and child health care, and fertility practices yielded some of the greatest gains. However, given differences in context and baseline status of some countries, the relative contribution of various components and the percentage explained by decomposition varied somewhat. In addition, the impact of food security and improved dietary practices (including complementary feeding practices) could be understated in our findings due to limitations in data availability and quality. Lastly, accurate serial coverage data for most direct health sector nutrition interventions (e.g., supplementation) were generally lacking. Taken together, all such observational analyses are subject to ecological fallacies, and hence the relative contribution of various factors to improvements in stunting must be viewed as illustrative and associational rather than deterministic. Additionally, this study did not discuss findings from control or counterfactual countries (i.e., those that have not made significant national stunting reduction) though a second phase of the exemplars project is currently examining large-population countries (India, Pakistan, and Nigeria) with the aim of assessing these subnational trends and policy environments more robustly. The quality of anthropometry measures continues to be a challenges in survey datasets; we have examined key metrics of height, weight, and age quality in exemplar country survey datasets (**Supplemental Table 2**) and found no major issues in missingness, response mismatch, or digit preference in the included surveys.

The improved classification of nutrition actions presented in this article will allow countries to effectively plan, prioritize, and invest in strategies that will lead to the intended population-level nutrition-related outcomes. Given the complex and multicausal nature of suboptimal growth and development, we generally strive for multisectoral approaches. However, clearly demarcating responsibilities by sectors to deliver on their core sectoral mandates with an equity lens also has strong potential to build an enabling environment for nutrition at the household level. Building better action and accountability in terms of resources, finances, leadership, and other factors to enable this can accelerate gains in stunting. It will be critical not to lose the momentum that has been behind the recent global focus on nutrition; for this, we emphasize the importance of and need for strong governance, supportive sectoral strategies, and direct and indirect health interventions for improving nutrition outcomes.

### Future analyses

These types of holistic analyses can useful to gain a better understanding of country context and nutrition evolution, and will inform future programmatic choices. We suggest that our alternate framework of organizing nutrition interventions as direct/indirect and inside/outside the health sector (in place of nutrition-specific and -sensitive) should be considered when mapping child stunting causal pathways and planning interventions and strategies to accelerate stunting reduction to achieve the 2030 SDGs. Our road map of diagnostics coupled with intervention prioritization could provide a valuable foundation for stunting reduction investment cases at national or regional scales. In addition, we call for a better coordination of the various efforts to understand drivers of nutrition change in a country, and accelerated efforts to undertake these analyses where they have not been done.

### Improving data

Many aspects of the analysis are dependent on the proxy data that we have on hand as opposed to direct measures of constructs. To illustrate, the interface of maternal and newborn nutrition and its relation to epigenetic factors and fetal growth is well recognized (27). However, data on important indicators such as maternal prepregnancy BMI, weight gain during pregnancy, and, importantly, gestational age and birthweight are not available. Our exercises ended up deploying a range of proxy indicators in an attempt to better understand the link between maternal nutrition, intrauterine growth, birth weight, and infant growth faltering. With the global drive to improve data capture around birth weight (28), this might improve over time. The same can be stated around disease management, maternal and child diet quality, and coverage of nutrition interventions, where existing survey data are presently insufficient to adequately assess their relative contributions to change over time. Furthermore, existing information on WASH coverage and behaviors does not include important information on frequency, quality, and fidelity of interventions and use, which could be rate-limiting factors in recent trials that have failed to show benefits of hand washing and sanitation interventions alone on linear growth in children (29). Taking these learnings around data collection and applying them to future studies and programs will be critical. In addition, recent consensus on coverage indicators for evidence-based nutrition interventions provides an important opportunity to improve data for future analyses and programmatic and policy direction (30).

Our exercise was also somewhat limited in that we did not have sufficient longitudinal information on population micronutrient status as well as concurrent stunting and wasting (17, 31). Future studies should be able to identify exemplar countries as those that have seen reductions across all the dimensions of childhood undernutrition. Finally, we did not take into account the concomitant time trends of childhood and adolescent overweight and obesity in the exemplar countries. True exemplars could well be the subset of countries that have seen improvements in undernutrition without the unintended consequences of overweight and obesity at population level, a phenomenon now being observed in most LMICs (32). In addition to known associations between overweight/obesity and the food environment (33), a few evaluations have shown that food distribution and school feeding programs themselves, intended to address undernutrition, could contribute to the growth in child overweight and obesity (34, 35). Future studies should include these complexities in the mix of determining true exemplars, especially in regions of the world with a growing double burden of malnutrition.

### Policy and programmatic way forward

As highlighted throughout this supplement, the specific drivers of reductions in national-level stunting prevalence for individual countries vary. However, there exist a set of key determinants that must be addressed with policy change and programmatic action to drive further progress on reducing stunting, optimizing child growth and development, and, ultimately, achieving 2030 SDG health and nutrition targets. The opportunities and ambitions to scale up actions in high-burden countries must be matched with financing needs, as assessed by the World Bank, and optimizing available resources (Optima Nutrition) so as to manage expectations of impact. Other collections of country case studies on the key drivers of improved nutrition, such as the “Stories of change in nutrition” (20) and “Stop stunting in South Asia” (36), have highlighted the particular importance of strong leadership, political and institutional commitment to change, and strategic investments targeting the most vulnerable and addressing the biggest risk factors for child growth. Our findings support and concur on the critical importance of these enablers to sustain improvements in child undernutrition. We also stress the vital importance of multisectoral action plans for targeting undernutrition, though the varying importance of sectors will differ based on context. The Scaling Up Nutrition (SUN) movement provides a collaborative and multisectoral framework for governments to commit to ending malnutrition in all its forms (37), but it is important that these plans align with the evidence base. The WHO has also compiled a list of evidence-based Essential Nutrition Actions (38) that countries can use to address the immediate determinants of malnutrition, taking into account the local context and progress toward achieving global nutrition targets. At the tail end of the decade of nutrition (39), acceleration of activities within nutrition programs is urgently needed for countries to reach the targets set by the WHA and the SDGs. Our suggested sequence of actions offers a pragmatic evidence-based approach to doing so. Notwithstanding the need for more information and research, we now know enough to move to action at scale, the time for which is now.

## Supplementary Material

nqaa153_Supplemental_File

## Notes

This study was funded by a grant to the Centre for Global Child Health from Gates Ventures.

The funders had no role in the design, implementation, analysis, and interpretation of the data.

Data described in the manuscript, code book, and analytic code will be made available upon request pending application.

Publication costs for this supplement were defrayed in part by the payment of page charges. The opinions expressed in this publication are those of the authors and are not attributable to the sponsors or the publisher, Editor, or Editorial Board of *The American Journal of Clinical Nutrition*.

Supplemental Figure 1 and Supplemental Tables 1 and 2 are available from the “Supplementary data” link in the online posting of the article and from the same link in the online table of contents at https://academic.oup.com/ajcn/.

Abbreviations used: HAZ, height-for-age *z*-score; LMIC, low- and middle-income countries; SDG, Sustainable Development Goal; WASH, water, sanitation, and hygiene; WHA, World Health Assembly.
