# Supplementary material for: How countries can reduce child stunting at scale: lessons from exemplar countries
Source: Am J Clin Nutr. 2020 Jul 21;112(Suppl 2):894S–904S. doi: 10.1093/ajcn/nqaa153 (PMC7487427; doi:10.1093/ajcn/nqaa153)
Supplement: nqaa153_Supplemental_File [file nqaa153_supplemental_file.docx]

**Supplementary Figure 1a: HAZ kernel density plots in Exemplar countries (start year)**


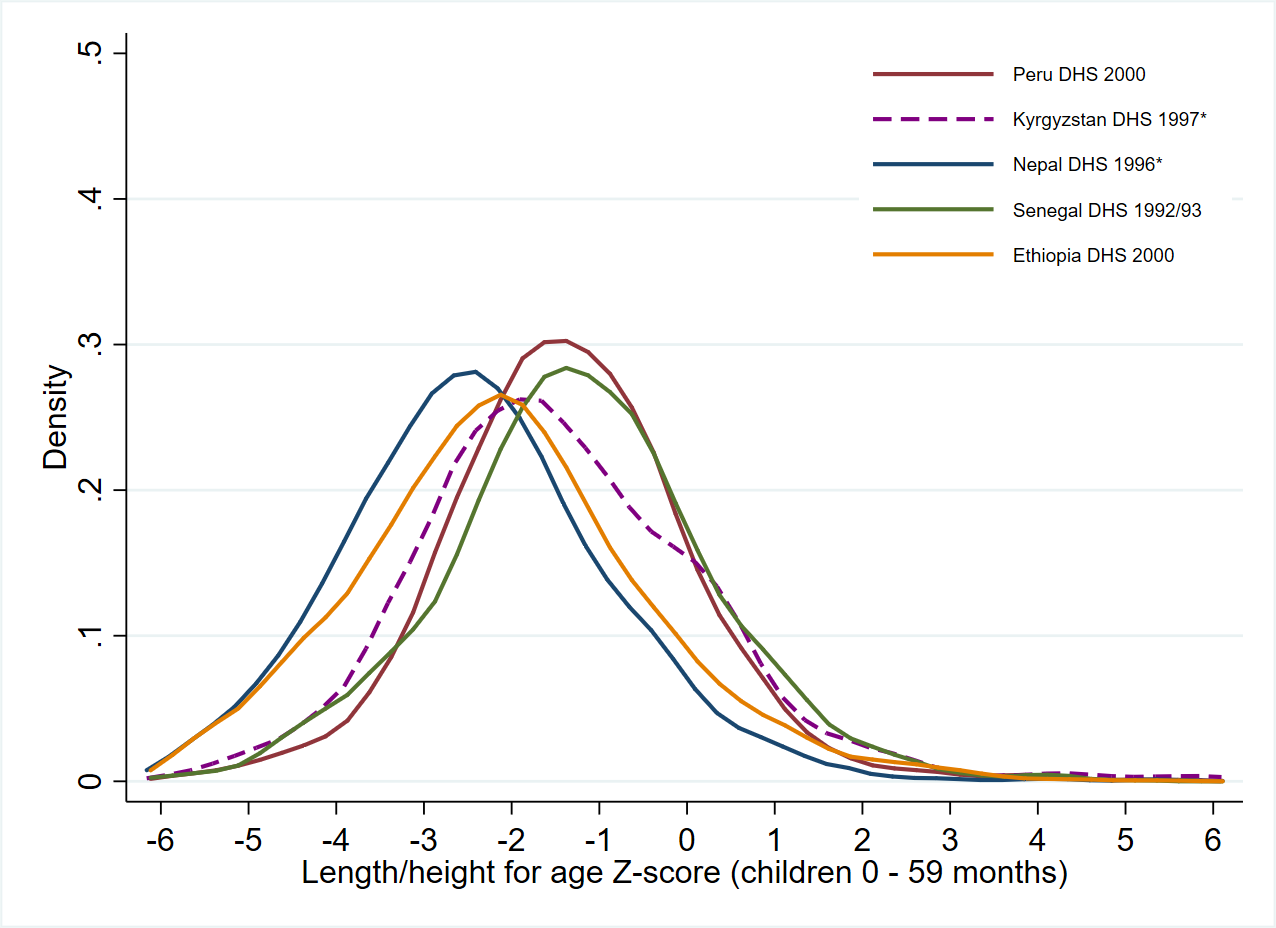


**Supplementary Figure 1b: HAZ kernel density plots in Exemplar countries (end year)**

**
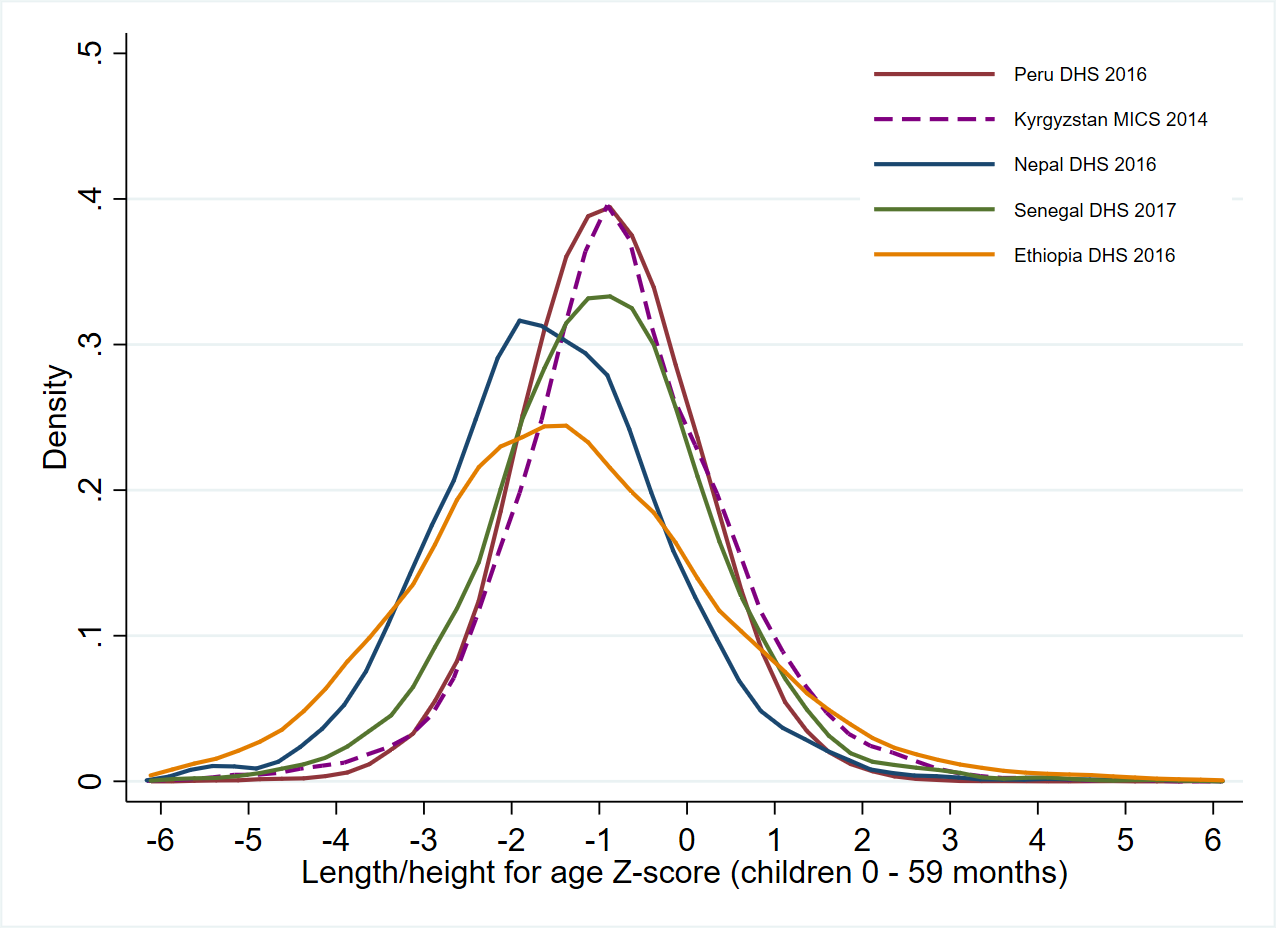
**

**Supplementary Table 1: Mean and Kurtosis for HAZ kernel density plots in Exemplar countries**

| Ethiopia |  |  | Kyrgyz Republic |  |  | Nepal |  |  | Peru |  |  | Senegal |  |  |
| --- | --- | --- | --- | --- | --- | --- | --- | --- | --- | --- | --- | --- | --- | --- |
|  | Mean | Kurtosis |  | Mean | Kurtosis |  | Mean | Kurtosis |  | Mean | Kurtosis |  | Mean | Kurtosis |
| 2000 | -2.24 | 3.65 | 1997 | -1.48 | 4.39 | 1996 | -2.41 | 3.92 | 2000 | -1.24 | 4.37 | 1992 93 | -1.25 | 3.56 |
| 2005 | -1.90 | 3.76 | 2005 | -0.62 | 4.22 | 2001 | -2.19 | 3.68 | 2007 08 | -1.21 | 3.90 | 2000 | -1.05 | 4.16 |
| 2011 | -1.68 | 3.68 | 2012 | -0.79 | 4.47 | 2006 | -1.92 | 3.38 | 2016 | -0.84 | 3.44 | 2005 | -0.85 | 4.14 |
| 2016 | -1.44 | 3.69 | 2014 | -0.71 | 4.45 | 2011 | -1.66 | 3.42 |  |  |  | 2017 | -0.85 | 4.10 |
|  |  |  |  |  |  | 2016 | -1.52 | 4.40 |  |  |  |  |  |  |

**Supplementary Table 2: Data quality indicators for Nepal, Kyrgyz Republic, Ethiopia, Senegal, and Peru based on WHO Anthro Survey Analyser.**

| **Country** | **Year** | **Total U5 population** | **U5 Population with anthropometric measures** | **Missing (height)** | **Mismatch length/height measurement position** | **Digit preference (height)** |
| --- | --- | --- | --- | --- | --- | --- |
| **Nepal** | 1996 | 4417 | 4083 | 6.6% (10% in eastern and midwestern regions) | <3% | Slight preference for .0 (13%) and .5 (13%) |
|  | 2001 | 6931 | 6353 | 1.6% | <2% | Preference for .0 (15%) and .5 (15%) |
|  | 2006 | 5783 | 5378 | 1.8% | <2% | No digit preference |
|  | 2011 | 5306 | 2392 | 1.4% | <2% | Preference for .0 (15%) and .5 (13%) |
|  | 2014 | 5663 | 5349 | 2.9% | <2% | No digit preference overall; varied depending on region (e.g. preference for .3 close to 30% in Western Mountain) |
|  | 2016 | 5031 | 2404 | 1.2% | <3% | No digit preference overall; some variation by region (but <20%) |
| **Kyrgyz Republic** | 1997 | 1127 | 1068 | 7.2% (10% in Bishkek) | 13% for 24-35 mo | Preference for .0 (46%) and .5 (22%); 50-60% in East and South |
|  | 2006 | 2987 | 2987 | 1.9% | 6% for 12-23 mo | Preference for .0 (57%); >60% and up to 80% in some regions |
|  | 2012 | 4363 | 4190 | 2.9% (10% in Bishkek) | 41% for 12-23 mo; 18% for 24-35 mo; 14% overall | Preference for .0 (22%) and .5 (15%); 20-60% in some regions |
|  | 2014 | 4611 | 4577 | 2.3% (10% in Chui) | 11% for 12-23 mo | No digit preference overall; some preference by region (e.g. 50% .0 in Issyk-Kul) |
| **Ethiopia** | 2000 | 10873 | 9218 | 1.7% (7% in Harari) | 15% for 12-23 mo; 13% for 24-35 mo; 6% overall | Preference for .0 (18%) and .5 (16%); <20% in some regions |
|  | 2005 | 9861 | 4455 | 5.9% (12.6% in Gambela, 10% in Addis and Somali) | 30% for children 12-23 mo; 12% for 24-35 mo; 10% overall | Preference for .0 (25%) and .5 (18%); 30-40% in some regions |
|  | 2011 | 11654 | 10480 | 5.7% (13% in Harari, 12% in Somali, 10% in Addis) | 43% for 24-35 mo; 14% for 36-47 mo; 15% overall | No digit preference overall; preference for .0 and .5 in some regions (e.g. >20% .0 in Harari) |
|  | 2016 | 10627 | 9685 | 6.5% (15% in Harari, 14% in Dire Dawa, 10% in Somali) | 17% for 12-23 mo | Preference for .0 (17%) and .5 (16%); >20% in some regions |
| **Senegal** | 1992 | 5645 | 5068 | 8% | 23% for 12-23 months; 14% for 24-35 months | None overall; .0 preference in North East (~17%) |
|  | 2000 | 9059 | 8794 | 3.1% (7% in Dakar) | 42% for 12-23 mo; 12% overall | Preference for .0 (23%) and .5 (17%); 20-40% for some regions (e.g. Kolda) |
|  | 2005 | 10944 | 3168 | 7% (17.5% in Matam, 13.3% in Saint-Louis, 12.3% in Louga) | 35% in 12-23 months; 12% overall | .0 and .5 preference overall (~16%); similar results in Dakar, Kolda, Tambacounda, Zuguinchor. |
|  | 2017 | 12185 | 11127 | <3% missing | 3% overall | No preference overall; .0 preference in Dakar, .5 preference in Kaolack |
| **Peru** | 2000 | 13697 | 12901 | ~8% missing | 11.5% in 12-23 months; <4% overall | .0 preference overall (~18%); similar in Amazonas, Arequipa, Cajamarca, Hauncavelica, Huanuco, Ica, Lima, Pasco |
|  | 2007/8 | 17189 | 11423 | ~8% missing | Negligible (<.5%) | .0 preference overall (~14%); .0 preference in Huanuco, La Libertad, Pasco, Tumbes; .0 and .5 preference in Cajamarca, Junin |
|  | 2016 | 23780 | 22669 | <4% missing | Negligible (<0.001%) | No preference overall; .0 preference in Arequipa, Huanuco, .4 preference in Tumbes |
